# Role of Bone Marrow-Derived Stem Cells in Polyps Development in Mice with Apc^Min/+^ Mutation

**DOI:** 10.1155/2015/354193

**Published:** 2015-06-16

**Authors:** Michele Barone, Maria Principia Scavo, Raffaele Licinio, Michele Piombino, Nicola De Tullio, Rosanna Mallamaci, Alfredo Di Leo

**Affiliations:** ^1^Gastroenterology Unit, Department of Emergency and Organ Transplantation, University of Bari, Piazza G. Cesare 11, 70124 Bari, Italy; ^2^Methodist Research Institute, 6670 Bertner Avenue, Houston, TX 77030, USA; ^3^Radiotherapy Unit, Diagnostic Imaging Department, Polyclinic Hospital, Piazza G. Cesare 11, 70124 Bari, Italy; ^4^Department of Bioscience, Biotechnology and Biopharmaceutics, University of Bari, Via E. Orabona 4, 70124 Bari, Italy

## Abstract

We explored the hypothesis that an altered microenvironment (intestinal adenomatous polyp) could modify the differentiation program of bone marrow-derived stem cells (BMSCs), involving them in colon carcinogenesis. Sublethally irradiated 8-week-old female Apc^Min/+^ mice were transplanted with bone marrow (BM) cells obtained from either male age-matched Apc^Min/+^ (Apc-Tx-Apc) or wild type (WT) (WT-Tx-Apc) mice. At 4 and 7 weeks after transplantation, BM-derived colonocytes were recognized by colocalization of Y-chromosome and Cdx2 protein (specific colonocyte marker). Polyp number, volume, and grade of dysplasia were not influenced by irradiation/transplantation procedures since they were similar in both untreated female Apc^Min/+^ and Apc-Tx-Apc mice. At 4 and 7 weeks after transplantation, a progressive significant reduction of polyp number and volume was observed in WT-Tx-Apc mice. Moreover, the number of WT-Tx-Apc mice with a high-grade dysplastic polyps significantly decreased as compared to Apc-Tx-Apc mice. Finally, at 4 and 7 weeks after transplantation, WT-Tx-Apc mice showed a progressive significant increase of Y+/Cdx2+ cells in “normal” mucosa, whereas, in the adenomatous tissue, Y+/Cdx2+ cells remained substantially unvaried. Our findings demonstrate that WT BMSCs do not participate in polyp development but rather inhibit their growth. The substitution of genotypically altered colonocytes with Y+/Cdx2+ cells probably contributes to this process.

## 1. Introduction 

Different concepts support the fact that cancer may arise from resident stem cells: they are able to proliferate and can accumulate DNA mutations thanks to their sufficiently long life span [1]; among the cell population constituting a tumour, the ability to maintain tumour growth specifically resides in a restricted cell population [2, 3]; the existence of* cancer stem cells* has been demonstrated in several human solid cancers including colon cancer [4–7].

More recently, some studies have focused on the possibility that solid tumours may arise from bone marrow-derived stem cells (BMSCs). In fact, Houghton et al. have demonstrated the participation of BMSCs in gastric cancer, a finding confirmed by Avital et al. in other human solid organ cancers [8, 9]. On the other hand, Cogle et al. [10] suggested that BMSCs and their progeny may be incorporated within cancer tissues, but their involvement is sporadic and may be due to developmental mimicry. Finally, we have found that BMSCs do not participate in liver cancer development using an experimental model of hepatocarcinoma [11].

Based on the speculation that BMSCs could be directly involved in colon carcinogenesis as previously demonstrated in other human solid organ cancers [8, 9], we explored the hypothesis that an altered microenvironment, represented by adenomatous polyps, could facilitate the recruitment of bone marrow-derived stem cells (BMSCs) modifying their differentiation program. Such a condition would lead these BMSCs towards the carcinogenetic process.

To verify this hypothesis, we used a transgenic mouse model with Apc mutation (Apc^Min/+^). This model mimics the condition observed in humans affected by familial adenomatous polyposis (FAP) and is widely used for colorectal cancer (CRC) studies [12, 13].

In our experimental conditions we sublethally irradiated 8-week-old female Apc^Min/+^ transgenic mice, transplanted these animals with bone marrow cells (BMCs) obtained from male age-matched, wild type (WT) mice, and followed the fate of BMCs through the detection of the Y-chromosome. Finally, to characterize the BM-derived cells within the colon tissue we used the Cdx2 protein, which is considered a marker for intestinal cell lineage [14].

The novelty of the current study is based on the use of BM-derived cells without a genetic predisposition to cancer (from wild type animals) to validate the hypothesis of an involvement of healthy BMSCs in colon carcinogenesis by a locally altered tissue microenvironment.

## 2. Materials and Methods

### 2.1. Animals

For this study female and male mice with Apc mutation (Apc^Min/+^), as well as male wild type (WT) syngeneic animals (C57BL/6J), were obtained from Charles River (Calco, Italy). Upon arrival, all mice were kept in temperature-, air-, and light-controlled (light on from 7 AM to 7 PM) conditions and received water* ad libitum* and either a standard mouse diet (WT) or a high-fat low-fiber diet (Apc^Min/+^) [12]. All animals received humane care according to the criteria outlined in the Guide for the Care and Use of Laboratory Animals and the study was authorized by the local Ethical Committee.

### 2.2. Bone Marrow Transplantation and Study Design

Bone marrow (BM) transplantation was performed using a modification of the technique previously described [11]. Briefly, female Apc^Min/+^ mice were sublethally irradiated (800 rad) and transplanted with bone marrow cells (BMCs) obtained from either male WT mice (WT-Tx-Apc) or male Apc^Min/+^ mice (Apc-Tx-Apc) within 4–6 hours. To prepare BMCs, male WT or Apc^Min/+^ mice were sacrificed by cervical dislocation and the hind limbs, removed to collect BM, were flushed with Minimum Essential Medium (MEM) + 5% Fetal Bovine Serum (FCS) from the medullary cavities of the tibias and femurs using a 27 G needle. Then, cells were centrifuged at 1000 g for 4 min, at 4°C, and resuspended in an appropriate volume of medium in order to obtain a cell suspension with a concentration of 1 × 10^6^/100 *μ*L. Finally, 100 *μ*L of cell suspensions was injected in irradiated female mice through the tail vein.

This procedure has been previously used to demonstrate liver and other organs engraftment by BM-derived cells in studies regarding tissue reconstitution or carcinogenesis [10, 11, 15–17].

Our study design included five groups: group A consisted of 6 female Apc^Min/+^ transgenic mice that did not receive any treatment and were sacrificed at 12 weeks of age; group B consisted of 6 female Apc^Min/+^ transgenic mice that, at the age of 8 weeks, underwent bone marrow (BM) transplantation using as donors age-matched male Apc^Min/+^ mice and were sacrificed at 12 weeks of age; group C consisted of 10 female Apc^Min/+^ transgenic mice that, at the age of 8 weeks, underwent BM transplantation using as donors age-matched male WT mice and were sacrificed at 12 weeks of age; group D consisted of 6 female Apc^Min/+^ transgenic mice that, at the age of 8 weeks, underwent BM transplantation using as donors age-matched male Apc^Min/+^ mice and were sacrificed at 15 weeks of age; group E consisted of 10 female Apc^Min/+^ transgenic mice that, at the age of 8 weeks, underwent BM transplantation using as donors age-matched male WT mice and were sacrificed at 15 weeks of age.

### 2.3. Histological Studies

The entire small bowel and colon were excised to assess polyp number and size/volume as previously described [12]. Briefly, the small intestine and the colon were cut along the mesenteric insertion, placed on a paper strip at 0–4°C, and analyzed trough a stereomicroscope at 3x magnification. The small intestine as well as colon specimens were fixed in 10% neutral buffered formalin for 24 hours and embedded in paraffin in a “Swiss roll” fashion, enabling the full intestinal tract to be microscopically examined on 4 *μ*m thick slices. Number, volume of intestinal polyps, and grade of dysplasia were assessed on haematoxylin-eosin (H-E) preparations. Stained sections were examined in a blinded fashion. As previously described [12], a three-tiered system (mild, moderate, and severe) was used for grading dysplasia. Briefly, mild dysplasia was considered as the presence of hyperplastic glands with minimal architectural distortion, no mucin-depleted cells and minimal decrease of goblet cells, slight increase of nuclear/cytoplasmic ratio, preservation of nuclear polarity, negligible stratification of nuclei, and undetectable mitotic figures; moderate dysplasia was considered as the presence of crowded hyperplastic glands with stratified nuclei, abundant mucin-depleted cells and consistent decrease of goblet cells, moderate increase of nuclear/cytoplasmic ratio, and slight alterations of nuclear/cytoplasmic ratio; severe dysplasia was considered as back-to-back architectural glandular arrangement, depletion of goblet cells, markedly increased nuclear/cytoplasmic ratio with evidence of pleomorphic nuclei, loss of nuclear polarity and evidence of multistratification of nuclei, and frequent and atypical mitotic figures [18].

Additional consecutive slides were used to evaluate the presence of the Y-chromosome in combination with Cdx2 by in situ hybridization studies and immunofluorescence. For bone marrow studies spine specimens underwent decalcification in Mielodec solution (EDTA, HC1 mixture) (Bioptica, Milano, Italy) for 6 hours, before undergoing dehydration and paraffin inclusion [19]. The percentage of Y-chromosome positive cells in the BM of transplanted mice was similar to that previously observed [11].

### 2.4. Fluorescence In Situ Hybridization (FISH) for the Y-Chromosome

FISH for Y-chromosome was performed using a modification of the technique previously described [11]. A probe obtained from ID Labs Biotechnology (Palo Alto, CA, USA) was used for the detection of the Y-chromosome. For our studies, 4 *μ*m thick slides mounted on 3-aminopropyl-triethoxysilane- (APTS-) treated slides were used. They were first deparaffinized, rehydrated, and washed in PBS. Then, after digestion with proteinase K (Roche Diagnostics, Mannheim, Germany), 500 *μ*g/mL, in Tris-HCl 100 mM/EDTA 50 mM, pH 8.0, for 25 minutes at 37°C, slides were washed with PBS and underwent denaturation with 70% Formamide in 2x saline sodium citrate (SSC) for 2 minutes at 72°C. Immediately after, liver slides were transferred in ice cold ethanol 70°, 85°, 100° for 2 minutes each and dried in an air jet. The Y-chromosome probe was treated for preannealing (10 minutes at 75°C and 1 hour at 37°C), before being added to the preheated sections. Then, specimens were covered, sealed with rubber cement, heated at 68°C for 10 minutes, and then incubated overnight at 37°C in a humidified chamber. The slides were then washed according to the protocol provided by ID Labs and gently dried and then FITC-Avidin (VECTOR laboratories Fluorescent Avidin Kit, Burlingame, CA, USA) was added after dilution in PBS (1 : 100) and incubated for 30 minutes at room temperature. Finally all slides were washed in PBS and TOPRO-3 was added (Invitrogen, Carlsbad, CA, USA) for nuclear counterstaining.

### 2.5. Immunofluorescence

Rehydrated consecutive histological colon sections were subjected to epitope retrieval procedures with microwave using a Citrate solution, at pH 6.0 (15 minutes at 750 watts, followed to PBS-TWEEN 0.5% for 25 minutes). Then, sections were transferred in ice cold ethanol 70°, 85°, 100° for 2 minutes each, and dried. The Y-chromosome probe was treated as described before and then incubated overnight at 37°C in a humidified chamber. After a washing in 2x SSC containing 0.1% Igepal and a second washing with 4x SSC containing 0.05% Tween-20, a solution with 2% BSA in 4x SSC for 30′ at room temperature was used as bloking solution. After three washes, sections were gently dried and then FITC-Avidin (Vector laboratories Fluorescent Avidin Kit, Burlingame, CA, USA) was added, after dilution in PBS (1 : 100), and incubated for 30 min at room temperature. Subsequently, sections were washed in PBS for three times. Finally, sections were incubated overnight, at 4°C, with primary antibodies against rabbit, Cdx2 (Cell Signaling Technology) using a 1 : 20 dilution.

The anti-rabbit Alexa 555 (Invitrogen, Carlsbad, CA, USA) was used for the immunodetection of Cdx2, diluted at 1 : 100, and incubated at room temperature for 30 minutes. All sections were counterstained with TOPRO-3 (Invitrogen, Carlsbad, CA, USA), diluted 1 : 5000, and cover slipped.

Colocalization of Cdx2 with the Y-chromosome was explored by confocal microscopy. We used FISH to label Y-chromosome, as previously illustrated, followed by Cdx2 immunodetection on the same slide. For immunofluorescence detection of Cdx2 we used the Vector Fluorescent Avidin kit (Vector Laboratories, Burlingame, CA, USA) and TOPRO-3 (Invitrogen, Carlsbad, CA, USA) as nuclear counterstain.

### 2.6. Microscopy and Image Capture

For light microscopy, a Leica (Wetzlar, Germany) DM RB microscope was used, equipped with an Olympus SP-350 digital camera. Image acquisitions were performed using OLYCAM/IAS software (ATZ, Bari, Italy). For confocal microscopy a Leica (Wetzlar, Germany) TCS-SP2 microscope was used, equipped with a laser Ar/Ar Krypton *λ* 488 (green, for the Y-chromosome), Gre/Neon *λ* 543 (red, for Cdx2), and He/Neon *λ* 633 (blue, for TOPRO). Images were collected and analyzed by Interactive LCS software (Leica, Wetzlar, Germany).

### 2.7. Cell Counting

The identification of the Y-chromosome positive cells was based on the presence of green spots within the circumference of the nucleus. The identification of the Cdx2 positive cells was based on the presence of red spots by immunohistochemistry. The number of labelled cells over the total of cells counted, that is, the percentage of labelled cells (Labelling Index = LI), was calculated in at least 10 randomly selected fields. A separate count was performed for normal and tumoral tissue. In age-matched male mice the efficiency of the Y-chromosome detection was 74.0 ± 10.1%.

### 2.8. Statistical Analysis

Continuous variables were expressed as means ± standard deviations and compared using either *t* test or ANOVA. When the single-factor ANOVA rejected the hypothesis of the mean equality among the groups, Tukey test was applied for a comparison of the means of the different groups. The criterion used for this comparison was a significance level *p* < 0.05. Dichotomous variables were expressed as percentages and compared with Chi-Squared test corrected by Fisher's exact test. A *p* value < 0.05 was considered statistically significant.

## 3. Results

### 3.1. Macroscopic Findings

**Figure 1:**
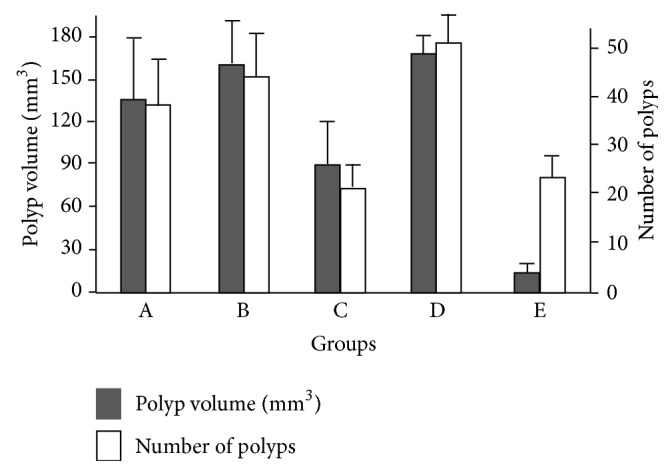
Polyp volume and number in untreated and transplanted Apc^Min/+^ female mice sacrificed at different time after bone marrow transplantation. The description of the different groups is reported in Section 2.2. Data represent the mean ± SD. Polyp volume and number resulted significantly different among the 5 groups by ANOVA (*p* < 0.005). Polyp volume in A = B = D ≠ Group C ≠ Group E by Tukey test. Polyp number in A = B = D ≠ Group C = E by Tukey test.

As shown in Figure 1, at 12 weeks of age, untreated female Apc^Min/+^ mice (group A) showed a number and volume of polyps similar to those observed in 12-week-old female Apc^Min/+^ mice transplanted at 8 weeks of age with BM cells obtained from age-matched male Apc^Min/+^ mice (group B, control) (37.5 ± 9.0 versus 42.8 ± 11.0 and 145.8 ± 35.5 mm^3^ versus 162.0 ± 40.2 mm^3^, resp.). On the other hand, 12-week-old female Apc^Min/+^ mice transplanted at 8 weeks of age with BM cells obtained from age-matched male WT mice (group C) showed a significant reduction of the number (21.6 ± 5.2 versus 42.8 ± 11.0) and the volume (89.9 ± 30.3 mm^3^ versus 162.0 ± 40.2 mm^3^) of polyps as compared to controls (group B) (*p* < 0.005).

Female 15-week-old Apc^Min/+^ mice transplanted at 8 weeks of age with BM cells obtained from age-matched male Apc^Min/+^ mice (group D) showed a moderate increased number (52.6 ± 12.0) and volume (172.2 ± 13.4 mm^3^) of polyps, as compared to group B mice, suggesting a progression of the disease. On the other hand, 15-week-old female Apc^Min/+^ mice transplanted at 8 weeks of age with BM cells obtained from male WT mice (group E) showed a polyp number that remained similar to that observed in group C but was significantly reduced as compared to group D (23.5 ± 4.2 versus 52.6 ± 12.0, *p* < 0.001), whereas polyp volume showed a further significant decrease as compared to group C (13.9 ± 5.2 versus 21.6 ± 5.2 mm^3^, *p* < 0.005) (Figure 1).

### 3.2. Evaluation of Dysplasia

**Figure 2:**
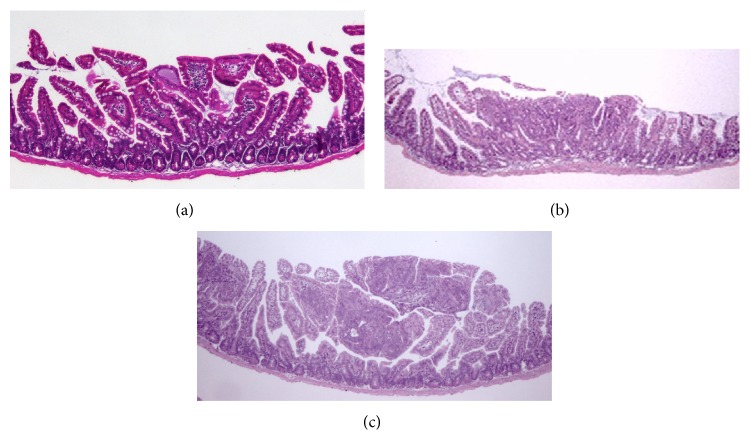
Histological aspects of adenomatous mucosa. H-E sections from polyps with mild (a), moderate (b), and severe (c) dysplasia.

Low, moderate, and highly dysplastic adenomatous polyps were observed in all animals (Figure 2). It is noteworthy that the number of polyps with high grade dysplasia was significantly reduced in group E as compared to group D mice (*p* = 0.034) (Table 1).

### 3.3. FISH and Immunohistochemical Studies

**Figure 3:**
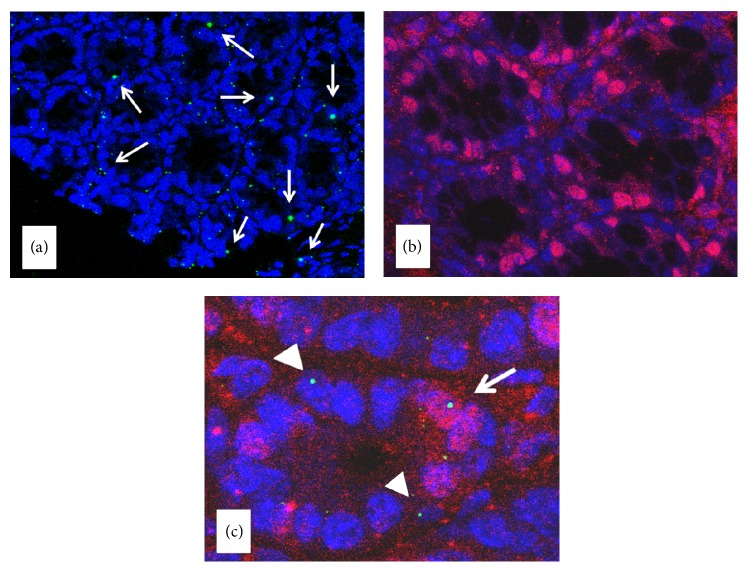
Y-chromosome and CDX-2 distribution in the “normal” mucosa and their colocalization in the nucleus. (a) Y-chromosome, identified by the green spot; (b) Cdx2, identified by the fuchsia intranuclear colour; (c) Y-chromosome, identified by the green spot, not colocalized with Cdx2 (arrow head). Y-chromosome colocalized with Cdx2, identified by the fuchsia intranuclear colour (arrows).

In order to interpret the macroscopic results regarding the variation of polyp size and number, we evaluated the presence of BM-derived cell in the “normal” and adenomatous tissue using Y-chromosome (Figure 3(a)). In addition, using the Cdx2 as marker of epithelial cell differentiation, specific for colonocytes (Figure 3(b)), we characterize the phenotype of Y-chromosome positive cells in the colonic mucosa. Figure 3(c) shows the colocalization of Y-chromosome and Cdx2 in the nucleus of nonadenomatous colon mucosa from group C mice. Y-chromosome positive cells in the intestinal mucosa ranged from 10% to 40% of the entire epithelial cell population while Y-chromosome/Cdx2 positive cells were about 22 ± 5% of the Y-chromosome positive cells (data not shown).

**Figure 4:**
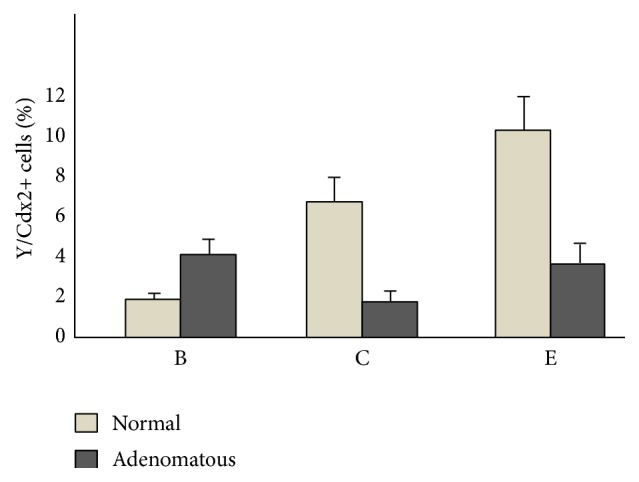
Y-chromosome/CDX-2 positive cells in “normal” and adenomatous tissue in female with Apc mutation transplanted mice. The description of the different groups is reported in Section 2. Data represent the mean ± SD. Y-chromosome/CDX-2 positive cells in the “normal” tissue resulted significantly different among the 3 groups, by ANOVA (*p* < 0.001). B ≠ C ≠ E by Tukey test. Positive cells in the adenomatous tissue resulted significantly different among the 3 groups, by ANOVA (*p* < 0.01). B = E ≠ C by Tukey test.

In Figure 4 the percentage of Y-chromosome/Cdx2 positive cells in “normal” and adenomatous tissue in transplanted Apc^Min/+^ female mice is reported. Our results demonstrate that at four weeks after transplantation, in group B, the Y/Cdx2 positive cells represented 1.9 ± 0.2% and 4.1% ± 0.6 of the cells in “normal” and adenomatous tissue, respectively. In group C, the percentage of Y/Cdx2 positive cells had an opposite distribution. In particular, they had a statistically significant increase in “normal” tissue and decrease in adenomatous tissue (6.8% ± 1.2 and 1.8% ± 0.3, resp.). Even more interesting is the distribution of Y/Cdx2 positive cells three weeks later (i.e., 7 weeks after transplantation) in group E. In fact, in these mice the percentage of Y/Cdx2 positive cells significantly grew up to 10.3% ± 2.1 and 3.9% ± 0.5 in “normal” and adenomatous tissue, respectively. Finally, also the distribution of Y/Cdx2 positive cells was different in the two groups. In fact, while in group B positive cells were prevalently localized in the lower/bottom part of the intestinal glands, in groups C and E, they were found essentially in the surface epithelium and in the middle/upper part of the intestinal glands (data not shown).

## 4. Discussion

Colorectal cancer (CRC) mostly involves polyp formation, a condition considered as precancerous [20]. The risk of progression toward overt CRC seems to be related to the number and the size of polyps [21], two conditions that are greatly expressed in the Apc^Min/+^ mice model.

Our experimental setting explored the hypothesis that an altered microenvironment, represented by the adenomatous polyps, could modify the differentiation program of BMSCs causing their involvement in cancer development. For this purpose we followed the fate of BM cells obtained from WT male mice, transplanted in Apc^Min/+^ female mice, by tracking Y-chromosome and using Cdx2 as marker intestinal cell lineage [14].

In a previous study conducted by Cogle et al. [10] in intact Apc^Min/+^ female mice receiving BM cells with Apc^Min/+^ mutation from age-matched Apc^Min/+^ male mice, a striking lower number of colonic Y-chromosome positive cells were found, concluding that there was no participation of BMCs in colonic carcinogenesis. Our study presents two new aspects; first of all we evaluated the effect of WT BMCs on polyp growth not only once but at different time from transplantation, in order to catch the evolutionary aspect of the process. Secondly, in our experimental conditions, the percentage of Y-chromosome/Cdx2 positive cells in the colonic mucosa was evaluated by a different detection technique, which could explain our different results. In fact, we did not use as colonic cell marker cytokeratins, which are localized on the cell membrane and therefore can offer problems of colocalization with Y-chromosome, but Cdx2, an intracellular marker mostly localized in the nucleus, which excludes problems of false positivity [14]. In another mouse model, harbouring genetic marker elements, De Jong et al. [22] found a low percentage of the intestinal epithelium colonization by BM cells. However, while we used as a marker the Y-chromosome, which is a constitutively expressed gene, their method was based on genetic manipulations whose expression could be influenced by the organ/tissue considered.

To rule out the influence of irradiation/transplantation procedures on polyp development we evaluated the number and the volume of polyps in the intact Apc^Min/+^ mice (group A) and age-matched Apc^Min/+^ mice receiving male BM cells with Apc^Min/+^ mutation (group B) demonstrating that both parameters were similar in these two groups. In group B, the number of Y/Cdx2 positive cell count was about 2% and 4% in “normal” and adenomatous tissue, respectively, suggesting that there is a certain recruitment of BM cells in the colon, more evident in the more actively proliferating adenomatous tissue.

The transplantation of WT-BM cells determined a striking significant reduction (about 50%) of polyp number and volume in group C as compared to group B. This effect was even more evident in group E mice, that is, 7 weeks after transplantation. In fact, the inhibitory effect toward polyp development exerted by WT-BM cells determined a further reduction of polyp number (55%) and volume (92%) in group E as compared to age matched mice receiving an Apc^Min/+^-BM transplantation (group D). Finally, the number of mice with polyps showing severe dysplastic lesions was reduced in groups C and E, reaching statistical significance in the latter group.

To interpret this reduction of polyp number and volume we evaluated the number of Y-chromosome positive cells in “normal” and adenomatous tissue. In the “normal” tissue of group C mice, we found a 5-fold increase of Y/Cdx2 positive cells as compared to group B, which reached more than 10% of the colonic epithelial cell population, while in adenomatous tissue the number of Y/Cdx2 positive cells remained unchanged. In group E, we observed a further significant increase of Y/Cdx2 positive cells in both “normal” and adenomatous tissue. This phenomenon suggests that there is a tendency to substitute Apc^Min/+^ cells with healthy cells, reducing the probability of new polyp formation. However, we cannot exclude that the inhibitory effects on polyp development were also due to a modification of the resident stem cell niche by different BM cell lineages [23]. In fact, Y/Cdx2 positive cells represented only 22 ± 5% of the total number of Y-chromosome positive cells found in the colon tissue.

The data on Y-chromosome/Cdx2 colocalization in “normal” intestinal mucosa indicate that WT-BM cells recruited from the intestine give origin to a larger population than that generated by Apc^Min/+^-BM cells as demonstrated by their number in groups C and B, respectively, and by the fact that WT cells were found not only in the glands but also in the surface epithelium while the distribution of Y-chromosome+/Cdx2+/Apc^Min/+^ cells was limited to the bottom of the glands.

On the basis of the results on polyp size and number, and considering that in both “normal” and adenomatous tissue the number of Y/Cdx2 positive cells after WT-BM transplantation progressively increased, we can state that WT-BM cells do not participate in the neoplastic progression but, on the contrary, reduce polyp formation and especially polyp growth with an almost “curative” effect.

In conclusion, our findings exclude the hypothesis that the altered microenvironment represented by the adenomatous polyp could modify the differentiation program of BM-derived stem cells.

## Figures and Tables

**Table 1:** Distribution of high-grade (HG) dysplastic polyps among the different groups.

Group	Number of mice	Number of polyps	Number of mice with HG-dysplastic polyps
A	6	37.5 ± 9.0	5 (83%)
B	6	42.8 ± 11.0	5 (83%)
C	10	21.6 ± 5.2	5 (50%)
D	6	52.6 ± 12.0	6 (100%)^*∗*^
E	10	23.5 ± 4.2	4 (40%)^*∗*^

^*∗*^Group E versus group D *p* = 0.034 by Chi-squared.
